# Impact of pH-Responsive Cisplatin/Ribavirin-Loaded Monodispersed Magnetic Silica Nanocomposite on A549 Lung Cancer Cells

**DOI:** 10.3390/pharmaceutics17050631

**Published:** 2025-05-09

**Authors:** Dana Almohazey, Vijaya Ravinayagam, Hatim Dafalla, Rabindran Jermy Balasamy

**Affiliations:** 1 Department of Stem Cell Research, Institute for Research and Medical Consultations, Imam Abdulrahman Bin Faisal University, Dammam 31441, Saudi Arabia; daaalmohazey@iau.edu.sa; 2 Deanship of Scientific Research, Imam Abdulrahman Bin Faisal University, Dammam 31441, Saudi Arabia; vrnayagam@iau.edu.sa; 3 Core Research Facilitites, King Fahd University of Petroleum and Minerals, Dhahran 31261, Saudi Arabia; dmhatim@kfupm.edu.sa; 4 Department of Nano-Medicine Research, Institute for Research and Medical Consultations, Imam Abdulrahman Bin Faisal University, Dammam 31441, Saudi Arabia

**Keywords:** monodispersed spherical silica, SPIONs, multifunctional, cancer, antiviral, pulmonary drug delivery

## Abstract

**Background/Objectives**: Nanocarrier particle design for treating chronic pulmonary diseases presents several challenges, including anatomical and physiological barriers. Drug-repurposing technology using monodispersed spherical silica is one of the innovative ways to deliver drugs. In the present study, the anticancer potential of combinational cisplatin/ribavirin was explored for targeted lung cancer therapeutics. **Methods**: Monodispersed spherical silica (80 nm) capable of diffusing into the tracheal mucus region was chosen and doped with 10 wt% superparamagnetic iron oxide nanoparticles (SPIONs). Subsequently, it was wrapped with chitosan (Chi, 0.6 wt/vol%), functionalized with 5% wt/wt cisplatin (Cp)/ribavarin (Rib) and angiotensin-converting enzyme 2 (ACE-2) (1.0 μL/mL). Formulations are based on monodispersed spherical silica or halloysite and are termed as (S/MSSiO_2_/Chi/Cp/Rib) or (S/Hal/Chi/Cp/Rib), respectively. **Results**: X-ray diffraction (XRD) and diffuse reflectance UV-visible spectroscopy (DRS-UV-vis) analysis of S/MSSiO_2_/Chi/Cp/Rib confirmed the presence of SPION nanoclusters on the silica surface (45% coverage). The wrapping of chitosan on the silica was confirmed with a Fourier transformed infrared (FTIR) stretching band at 670 cm^−1^ and ascribed to the amide group of the polymer. The surface charge by zetasizer and saturation magnetization by vibrating sample magnetometer (VSM) were found to be −15.3 mV and 8.4 emu/g. The dialysis membrane technique was used to study the Cp and Rib release between the tumor microenvironment and normal pH ranges from 5.5 to 7.4. S/MSSiO_2_/Chi formulation demonstrated pH-responsive Cp and Rib at acidic pH (5.6) and normal pH (7.4). Cp and Rib showed release of ~27% and ~17% at pH 5.6, which decreases to ~14% and ~3.2% at pH 7.4, respectively. To assess the compatibility and cytotoxic effect of our nanocomposites, the cell viability assay (MTT) was conducted on cancer lung cells A549 and normal HEK293 cells. **Conclusions**: The study shows that the designed nanoformulations with multifunctional capabilities are able to diffuse into the lung cells bound with dual drugs and the ACE-2 receptor.

## 1. Introduction

Lung cancer is the leading cause of cancer-related deaths worldwide and accounts for approximately 1.8 million deaths annually, and is often diagnosed at advanced stages, with around 2.48 million new cases reported each year [1]. While smoking-related lung cancer remains the primary cause of these deaths, there has been a recent increase in lung cancer among non-smokers attributed to factors like genetic mutations, air pollution, and other environmental exposures [2]. Notably, among all types of cancers, lung cancer has one of the lowest survival rates (10–20%) with conventional chemotherapies; it is often associated with high recurrence rates and significant adverse side effects [3,4]. In recent years, drug repurposing has emerged as an attractive strategy, as it targets tumor sites, reduces drug dosage, overcomes drug resistance, and offers the convenience of utilizing FDA-approved drugs, thus significantly lowering research and development costs [5,6,7]. Nanotechnology-driven nanocarriers have demonstrated enhanced pharmacokinetic and pharmacodynamic properties without altering the molecular structure of the chemotherapeutic drugs. They facilitate passage through biological barriers, reduce toxicity to normal cells, and improve bioavailability [8,9,10]. Pulmonary route delivery offers several advantages for treating respiratory conditions, including rapid onset of therapeutic action, localized treatment of infections, and non-invasive administration. However, designing an effective nanocarrier for pulmonary disease is challenging due to the complex anatomical, physiological, and physicochemical characteristics of the lungs. Nanoparticle formulation design must take into account factors such as cellular uptake, particle deposition, the enhanced permeability and retention effect, as well as strategies to evade phagocytosis and mucociliary clearance [11]. For instance, designing nanoparticles to effectively target the airways (trachea, bronchi) and alveolar regions requires precise control over particle size and formulation. Particles with a diameter between 1 and 5 μm typically deposit in the alveolar region, while those smaller than 3 μm can reach the lower airways. Particles smaller than 150 nm are particularly advantageous due to slower lung clearance index and improved protein interactions [12]. Nanosized particles ranging from 60 to 300 nm, especially when formulated with polymer nanocomposites, have been reported to diffuse freely through tracheal mucus and penetrate respiratory mucus barriers [13]. Recently, monodispersed silica nanoparticles have attracted significant interest in the fields of optics, nanosensors, and biomedical applications due to their uniform particle size [14]. We have previously reported that copper ferrite impregnated on monodispersed silica exhibited cytotoxic effect on MCF-7 cells while maintaining high biocompatibility with HEK293 cells (95% cell viability). Furthermore, the impregnation of CeO_2_ in combination with cisplatin and curcumin on silica nanoparticles demonstrated enhanced cytotoxicity against MCF-7 cells [15,16]. Considering the various limitations of conventional anticancer drugs, including systemic toxicity and the development of drug resistance, researchers have increasingly explored the repurposing of antiviral agents for targeted cancer therapy. For example, the antiviral drug acyclovir has been reported to exert anticancer activity by downregulating aldehyde dehydrogenase in breast cancer cells [17]. Co-delivery of favipiravir with tamoxifen has been shown to inhibit tumor growth by inhibiting human telomerase reverse transcriptase in tamoxifen-resistant breast cancer cells [18]. Similarly, oseltamivir phosphate, a widely prescribed antiviral agent for influenza, has been reported to inhibit pancreatic cancer by suppressing sialidase activity [19,20]. Epidemiological data further suggest that users of oseltamivir phosphate exhibit reduced cancer incidence and mortality across multiple cancer types [20].

Lung cancer and viral infections are increasingly being recognized as interrelated, with certain viruses potentially contributing to the initiation and progression of the disease. Viruses such as human papillomavirus, Epstein–Barr virus, and cytomegalovirus have been detected in lung cancer tissues, suggesting a possible correlation [21,22,23]. Chronic inflammation resulting from prolonged viral infections can induce cellular damage and increase the likelihood of genetic mutations, a critical factor in carcinogenesis. Severe Acute Respiratory Syndrome (SARS), caused by the SARS coronavirus (SARS-CoV), is a respiratory illness that primarily affects the lungs and continues to pose a significant global health threat. Notably, emerging variants such as JN.1 have been associated with increased pulmonary infections and elevated mortality rates worldwide [24]. A recent study reported that approximately half of the lung cancer patients had COVID-19 without receiving a formal diagnosis [25,26]. Notably, the infection rate of SARS-CoV-2 among lung cancer patients was seven times higher than in the general population, and it was accompanied by elevated hospitalization and fatality rates exceeding 30% [27]. It is hypothesized that SARS-CoV-2 may play a critical role in promoting cancer stem cell development, primarily through the induction of a cytokine storm. The virus binds to angiotensin-converting enzyme 2 (ACE2), leading to the dysregulation of the renin–angiotensin–aldosterone system (RAAS). This dysregulation contributes to inflammation, oxidative stress, activation of proliferation signaling pathways, and tumor initiation and metastasis [28]. ACE2 serves as a key regulator of the RAAS and is essential for maintaining cardiovascular homeostatis, modulating acute inflammation, and managing autoimmune disorders [29,30,31]. Several studies have identified mechanistic overlaps between SARS-CoV-2 infection and cancer-related signaling pathways, including alterations in ACE-2 and TMPRSS2 expression (up-regulation), immune response, cytokine release, and a hypercoagulable state leading to a state of disequilibrium [32]. It has been determined that ACE2 was up-regulated in lung cancer patients, particularly in cases of lung adenocarcinoma and lung squamous cell carcinoma, and this upregulation is associated with reduced survival rates [33]. An example of shared signaling pathways between cancer and SARS-CoV-2 infection is the Janus kinase/signal transducer and activator of transcription (JAK-STAT) pathway [34]. This pathway, which plays a critical role in regulating immune responses, cell survival, and proliferation, has been well-established as a contributor to tumor initiation and progression. Dysfunction of the JAK-STAT pathway can result in enhanced cellular proliferation and tumor development [35]. Additionally, cytokines released in response to SARS-CoV-2 infection have been shown to activate the JAK-STAT pathway, leading to the recruitment of monocytes and neutrophils, thereby exacerbating inflammation [36,37]. Furthermore, a recent study investigating metabolic changes in lung cancer patients infected with SARS-CoV-2 reported decreased glutathione (GSH) levels and increased reactive oxygen species (ROS), which indicated heightened oxidative stress and cellular damage [38].

In this study, a novel nanoformulation was developed by doping superparamagnetic nanoparticles onto monodispersed silica (MSSiO_2_) for the co-delivery of cisplatin and ribavirin. The formulation was functionalized with ACE2 protein and coated with chitosan for potential application in lung cancer. Results demonstrated that the nanoformulation exhibited pH-responsive drug release and induced a dose-dependent cytotoxic effect on lung cells.

## 2. Materials and Methods

Superparamagnetic iron oxide nanoparticle (Fe_3_O_4_, 97%) with a particle size range of 50–100 nm and halloysite nanotube (Al_2_Si_2_O_5_(OH)_4_·2H_2_O) with a surface area of 64 m^2^/g were purchased from Sigma-Aldrich. Monodispersed spherical silica (SUPSIL STANDARD) was obtained from Superior Silica, LLC. (Chandler, AZ, USA). The anticancer drug cisplatin, antiviral drug ribavirin, lyophilized angiotensin converting enzyme-2 (ACE-2), and chitosan were also procured from Sigma-Aldrich. For cell culture studies, Dulbecco’s Modified Eagle Medium (DMEM), fetal bovine serum (FBS), 100X penicillin streptomycin, and 100X MEM non-essential amino acids (MEM NEAA) were obtained from Thermo Fisher Scientific (Waltham, MA, USA). Cell viability assay was performed using the 3-(4,5-dimethylthiazol-2-yl)-2,5-diphenyltetrazolium bromide (MTT) reagent (Thermo Fisher Scientific, Cat No. M6494). All reagents are manufactured in a cGMP-compliant facility.

### 2.1. Synthesis of SPIONs/MSSiO_2_ and SPIONs/Hal Nanocomposites

SPIONs/MSSiO_2_ and SPIONs/Hal nanocomposites were synthesized via a dry impregnation technique. Briefly, 0.5 g of SPIONs (10 wt%) was mixed with 5 g of MSSiO_2_ using a mortar and pestle. The resulting black powder was subsequently coated with chitosan (Chi) (0.6 wt/vol%). For the preparation of S/MSSiO_2_/Chi, 0.25 g of S/MSSiO_2_ was dispersed in 10 mL of chitosan solution and stirred vigorously at 800 rpm for 3 h. The mixture was then centrifuged at 14,000 rpm for 10 min, and the pellet was washed three times with 5 mL of deionized water to remove the physically adsorbed chitosan. The final product was dried at 45 °C for 12 h.

### 2.2. Preparation of S/MSSiO_2_/Chi/Cp/Rib (5% wt/wt)

Cisplatin (Cp) and ribavirin (Rib) were loaded onto the nanocomposite using normal saline solution (NSS). Briefly, 30 mg of Cp and 30 mg of Rib were mixed with 600 mg of S/MSSiO_2_/Chi in 10 mL of NSS under ice-cold conditions. The resulting mixture was centrifuged at 14,000 rpm to recover the functionalized nanocomposite. The pellet was washed with 5 mL of NSS to remove unbound drugs and subsequently dried at ambient temperature. The filterate was collected for quantitative analysis of Cp and Rib using UV-visible spectroscopy. For the preparation of S/Hal/Chi/Cp/Rib, an identical protocol was followed, substituting MSSiO_2_ with Hal as the nanocarrier material.

### 2.3. Preparation of S/MSSiO_2_/Chi/Cp/Rib/ACE-2

**Scheme 1:**
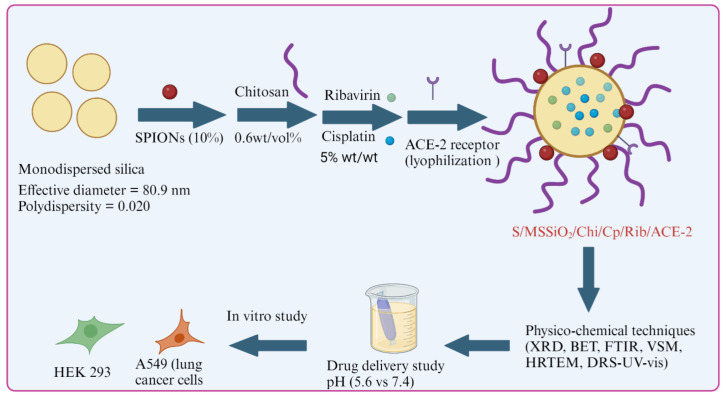
Fabrication of magnetic-monodispersed spherical silica loaded with cisplatin/ribavirin for potential treatment of lung cancer.

For ACE-2 functionalization, 1 μL of ACE-2 receptor was dissolved in 2 mL of NSS and stirred for 10 min. Then, 120 mg of S/MSSiO_2_/Ch/Cp/Rib mixture was dispersed in 1.5 ml distilled water and stirred overnight to ensure uniform mixing. The resulting suspension was lyophilized and stored at −20 °C until further use (Scheme 1).

### 2.4. Physicochemical Characterization

The crystalline and amorphous phases of drug-loaded nanocarriers were characterized by X-ray diffraction (XRD; Miniflex 600, Rigaku, Japan). The extent of surface occupation and pore changes after drug loadings was estimated using N_2_ adsorption–desorption isotherm (ASAP2020 plus, Micromeritics, Norcross, GA, USA). Briefly, 0.05 g of the sample was weighed, loaded into a quartz tube with a filler rod, and degassed at 50–150 °C for 24 h. After degassing, the sample was switched to the analysis section with standard multipoint Brunauer–Emmett–Teller measurement (30 Adsorption and 30 Desorption points) to evaluate the textural characteristics of MSSiO_2_, SPIONs/MSSiO_2_, and S/MSSiO_2_/Chi/Cp/Rib/ACE-2. The textural characterization of samples was provided in Table 1. The chemical environment of SPIONs impregnation into the silica matrix was examined by diffuse reflectance spectroscopy (DRS-UV; JASCO, V-750, Tokyo, Japan). Drug-silica interactions were confirmed by Fourier-transform infrared (FT-IR) spectroscopy (L160000A, Perkin Elmer, Waltham, MA, USA). Morphological and elemental analyses were conducted using scanning electron microscopy with energy dispersive X-ray spectroscopy (SEM-EDS; JSM-6610LV, JEOL, Tokyo, Japan) and Transmission electron microscopy (TEM; FEI, Morgagni 268, operated at 80 kV, Hillsboro, OR, USA).

### 2.5. In Vitro Drug Release Study

The dual release profile of nanoformulations was evaluated using the dialysis membrane diffusion method (molecular weight cut-off: 14,000 Da). Prior to the release study, calibration curves for Cp and Rib were established using standard solutions ranging from 5 to 30 μg/mL, measured at their respective maximum absorbance wavelength (λ_max)_. The linear regression of Cp was determined as y = 0.0096x + 0.0258 (R^2^ = 0.98), where y corresponds to absorbance and x corresponds to the Cp release concentration in µg per ml. The linear regression of Rib was determined as y = 0.0328x + 0.0069 (R^2^ = 0.99), where y corresponds to absorbance and x corresponds to the Rib release concentration in µg per ml. For the release study, 15 mg of nanoformulation was enclosed in the dialysis membrane and immersed in 25 mL of PBS at 37 °C. The release medium was maintained at two pH conditions: 5.6 and 7.4. At predetermined time intervals, 5 mL of release medium was withdrawn and replaced with an equal volume of fresh PBS to maintain sink conditions. The collected samples were analyzed by UV-visible spectroscopy. All experiments were conducted in duplicate.

### 2.6. Cell Culture and Treatment

To evaluate the cytotoxic effects of the synthesized nanocomposites, human lung carcinoma (A549) and the human embryonic kidney (HEK293) cell lines were employed. Both cell lines were from the American Type Culture Collection (ATCC). Cells were maintained in DMEM and supplemented with 10% HI-FBS, 1% Penicillin Streptomycin, and 1% MEM NEA at 37 °C and 5% CO_2_.

### 2.7. Cell Viability (MTT)

For the cell viability assay, 96-well plates were used with 20,000 cells/well. Cells were treated with our nanocomposites 24 h after seeding. Treatment conditions were as follows: S/MSSiO_2_/Chi, S/Hal/Chi, Cp, Rib, S/MSSiO_2_/Chi/Cp, S/Hal/Chi/Cp, S/MSSiO_2_/Chi/Cp/Rib, and S/Hal/Chi/Cp/Rib. The nanoformulation concentrations (except Cp and Rib) were as follows: 0.025, 0.05, 0.1, and 0.5 mg/mL. According to our drug loading experiment, there were 0.045 mg of Cp and Rib in 1 mg of our nanocomposites. Therefore, the treatment concentrations of free Cp and Rib were as follows: 0.001125, 0.00225, 0.0045, and 0.0225 mg/mL. In other words, if the treatment concentration of our nanocomposite was 0.5 mg/mL, then the treatment concentration for both Cp and Rib should be 0.0025 mg/mL. After 48 h of treatment, cells were washed, and the MTT solution (0.5 mg/mL) was added to each well for 3 h at 37 °C. After that, the formazan crystals were solubilized with 0.04 N HCl isopropyl alcohol. The change in color was detected using SYNERGY-neo2 BioTek ELISA reader at 570 nm. Each treatment condition was performed in duplicate (technical repeats) with three experimentally independent biological repeats (n = 3). Analysis was performed by comparing each condition with a no-treatment negative control. The percentage viability was calculated using the following formula:

Cell Viability (%) = (absorbance of treatment/absorbance of negative control) × 100.

### 2.8. Microscopic Examination

In order to assess cellular internalization, S/MSSiO_2_/Chi/Cp/Rib nanocomposite was functionalized with ACE-2 and Nile Red (NR). The modified nanocomposite was labeled as S/MSSiO_2_/Chi/Cp/Rib/ACE-2/NR. A549 and HEK293 cells were treated with both non-modified and modified conditions (S/MSSiO_2_/Chi/Cp/Rib and S/MSSiO_2_/Chi/Cp/Rib/ACE-2/NR) at 0.1 mg/mL for 24 h. Cells were then stained with Hoechst 33342 (Thermo Fisher Scientific, cat no. 62249) for 20 min, washed with PBS, and then viewed under the microscope. Zeiss LSM 700 (Jena, Germany) confocal microscope was used to capture the immunofluorescent images.

### 2.9. Statistics

The cell viability assay was performed in three experimentally independent biological repeats (n = 3). Statistical analysis was performed using Prism 9.2 software (GraphPad, La Jolla, CA, USA). The analysis was performed using two-way ANOVA with Dunnett’s post hoc test. Error bars ± S.E.M. Statistical significance and *p* values are listed in Table 2.

## 3. Results and Discussion

**Figure 1:**
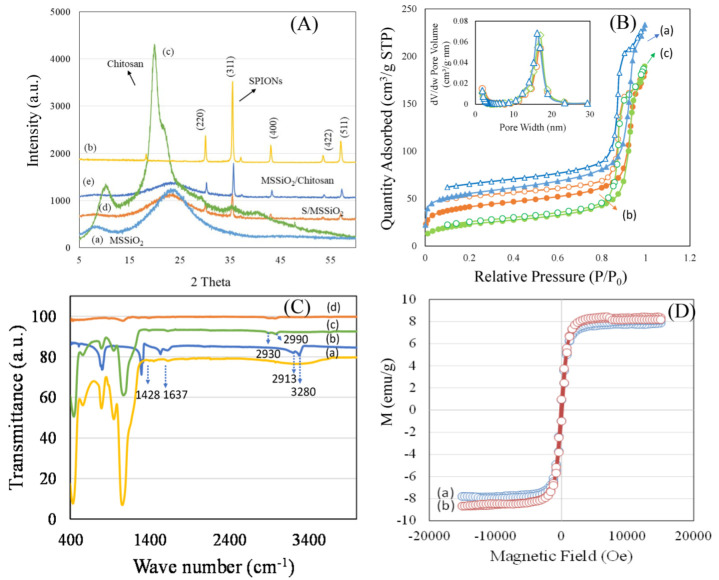
(**A**). X-ray diffraction (XRD) patterns of the following: (a) MSSiO_2_; (b) SPIONs; (c) chitosan; (d) SPIONs/MSSiO_2_; (e) SPIONs/MSSiO_2_/Chi. (**B**) Nitrogen adsorption–desorption isotherms of the following: (a) MSSiO_2_; (b) SPIONs/MSSiO_2_; (c) chitosan-wrapped SPIONs/MSSiO_2_. (**C**) FTIR spectra of the following: (a) MSSiO_2_; (b) cisplatin; (c) chitosan wrapped SPIONs/MSSiO_2_; (d) chitosan. (**D**) VSM analysis of the following: (a) SPIONs/MSSiO_2_; (b) SPIONs/Hal. Hollow symbols represent “Desorption”, solid symbols represent “Adsorption”.

XRD analysis was used to examine the crystalline and amorphous phases of SPIONs, drug-loaded formulations, and polymers. The XRD patterns reveal distinct peaks corresponding to SPIONs, silica (nanocarrier), chitosan, and the SPIONs-loaded silica–chitosan (S/MSSiO_2_/Chi) nanoformulation (Figure 1A(a–c)). Spherical silica exhibits a characteristic broad amorphous peak around 22.5° (Figure 1A(a)), while SPIONs display highly crystalline peaks between 15 and 60° (Figure 1A(b)). The parent chitosan shows a broad crystalline peak, which is attributed to hydrogen bonding (inter- and intramolecular forces) (Figure 1A(c)). The formation of a magnetic-silica nanocomposite is confirmed by the combined peaks corresponding to both spherical silica and crystalline SPIONs. Notably, the crystalline peaks of SPIONs are reduced, with peaks indexing at the (220), (311), (400), (422), (511), and (440) planes (Figure 1A(d,e)). In the case of S/MSSiO_2_/Chi formulation, the chitosan coating further diminishes the peak intensity, suggesting successful chitosan wrapping over the S/MSSiO_2_. Additionally, the crystalline peaks of chitosan disappear, indicating its structural transformation with formulation. The presence of high surface area of nanocarriers is expected to enhance the multifunctional capabilities of encapsulated drugs.

Nitrogen adsorption isotherm was employed to determine the textural changes, including surface area and pore characteristics of the nanocarrier and nanoformulation. Figure 1B(a–c) shows the N_2_ adsorption isotherm of the following: (a) MSSiO_2_; (b) S/MSSiO_2_; (c) S/MSSiO_2_/Chi/Cp/Rib/ACE-2. The textural properties were presented in Table 1. The parent MSSiO_2_ exhibited a type IV isotherm pattern, indicative of mesoporosity, with a high surface area of approximately 170 m^2^/g. The pore volume was measured at 0.35 cc/g, with pore diameter centered around 8.3 nm. In the case of SPIONs/MSSiO_2_, the surface area decreased to ~130 m^2^/g, indicating the occupation of SPIONs at the external surface of MSSiO_2_. Correspondingly, the pore volume decreased to 0.28 cm^3^/g. Following chitosan-wrapping and drug loading of SPIONs/MSSiO_2_ nanocarrier, a further reduction in surface area to 76 m^2^/g was observed. The quantity of nitrogen adsorbed decreased, while an increase in the pore diameter to 15 nm was observed. This indicates that the wrapping of chitosan occurs at the external pore walls of the SPIONs/MSS matrix, contributing to the increased pore diameter.

The interaction between Cp, chitosan, and nanocarrier was analyzed using FTIR spectroscopy. Figure 1C(a–d) show the FTIR spectra of the following: (a) MSSiO_2_; (b) Cp; (c) S/MSSiO_2_/chitosan; (d) chitosan. MSSiO_2_ exhibited typical functional moieties between 2970 and 3670 cm^−1^, corresponding to O-H and N-H stretching vibrations. The spectrum of Cp exhibited distinct peaks at 1557 and 3285 cm^−1^. In the case of S/MSSiO_2_/chitosan, the presence of absorption bands at about 2894 and 2998 cm−1 confirmed the chitosan wrapping onto the MSSiO_2_ surface. Furthermore, the attenuation of the O-H and N-H stretching bands at 3275 cm^−1^ supports the nanocomposite formation with silica. The magnetic properties of the nanoformulations were measured using a vibrating sample magnetometer (VSM). Figure 1D displays the hysteresis loops and saturation magnetization for the following: (a) S/MSSiO_2_; (b) S/Hal. Both samples demonstrated superparamagnetic properties, as evidenced by the absence of a hysteresis loop. This magnetic response is attributed to the presence of Fe^2+^ and Fe^3+^ species in Fe_3_O_4_, which contribute to the observed strong magnetism. The superparamagnetic characteristics indicate alignment of the magnetic moments with the applied magnetic field, facilitated by Fe^2+^/Fe^3+^ species and interactive dipole–dipole nature. Previous studies report a saturation magnetization of 67 emu/g for pristine SPIONs [39]. In the present study, saturation magnetization values of 8.4 emu/g and 8.2 emu/g were observed for SPIONs/MSSiO_2_ and SPIONs/Hal, respectively. This reduction in magnetization is likely due to decreased particle size and the formation of MSSiO_2_ and Hal-based nanocomposites. It has been reported that small nanocluster contribute toward the superparamagnetic property, while large nanocluster leads to ferromagnetism [40]. The superparamagnetic behavior observed here indicates the presence of small SPION nanoclusters on the surface of MSSiO_2_. Additionally, the noncollinear spin configuration at the silica interface could enhance the nanocomposite responsiveness to the external magnetic fields, offering further potential for magnetically guided applications.

**Figure 2:**
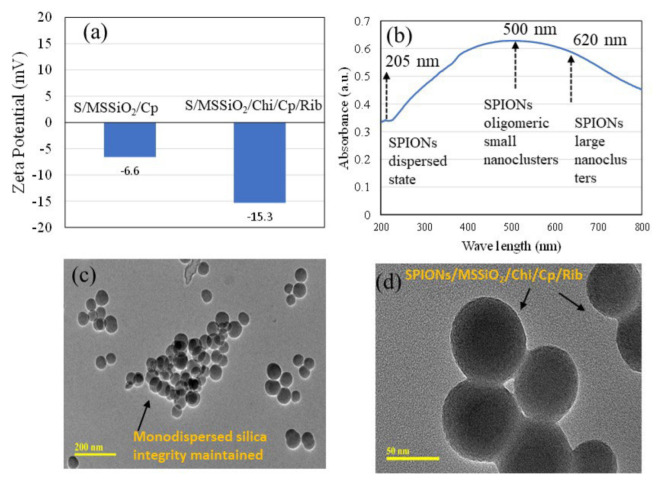
(**a**) Zeta potential of MSSiO_2_/Cp and MSSiO_2_/Chi/Cp/Rib. (**b**) Diffuse reflectance spectra of 10 wt% SPIONs/MSSiO_2_. (**c**,**d**) HRTEM images of MSSiO_2_/Chi/Cp/Rib at different magnification scales of 200 nm and 50 nm.

The zeta potential, coordination characteristics, and morphology of formulations were investigated using a zetasizer, DRS-UV-visible spectroscopy, and HRTEM (Figure 2a–d). The design of nanoparticles is crucial considering the physiological complexity of the lung, which involves various clearance mechanisms and barriers for eliminating the inhaled particles. Surface change and particle size have been shown to play a critical role in nanoparticle distribution and therapeutic efficacy. Notably, nanoparticles within the size range of 60–300 nm have been reported to penetrate tracheal mucus in an in vivo study [41]. In this study, Zetasizer characterization was performed to evaluate the surface charge and colloidal stability of formulations, including MSSiO_2_/Cp and MSSiO_2_/Chi/Cp/Rib. Prior to chitosan fabrication, the MSSiO_2_/Cp exhibited a relatively low negative zeta potential of −6.6 mV, indicating limited electrostatic stability. However, upon chitosan fabrication, the zeta potential of MSSiO_2_/Cp/Rib formulation increased to −15.3 mV (Figure 2a), suggesting enhanced colloidal stability. This increase is attributed to the cationic nature of chitosan, which modifies the surface potential and improves the overall stability of the nanocarrier system.

The coordination environment of SPIONs on the MSSiO_2_ was investigated using DRS-UV-Vis spectroscopy. The presence of a weak absorption peak at about 220 nm shows the tetrahedrally coordinated ferric species (Fe^3+^), indicating their dispersed state on the MSSiO_2_ surface. The appearance of a band maximum at 500 nm is indicative of the formation of oligomeric SPION nanoclusters, while the broadening of the absorption band above 500 nm suggests the presence of larger SPION clusters (Figure 2b). HRTEM analysis was employed to examine the morphology and particle size of the MSSiO_2_/Chi/Cp/Rib formulation at 200 nm and 50 nm, respectively. The analysis confirmed the retainment of monodispersity, with silica nanoparticles exhibiting a consistent size of approximately 80 nm (Figure 2c,d). The impregnation of SPIONs, functionalization with Cp and Rib, and wrapping with chitosan induced only minor changes in particle morphology, demonstrating that the structural integrity of MSSiO_2_ was largely maintained. This suggests the potential of the engineered monodispersed spherical silica-based nanocarrier for potential site-specific delivery to the lung.

**Figure 3:**
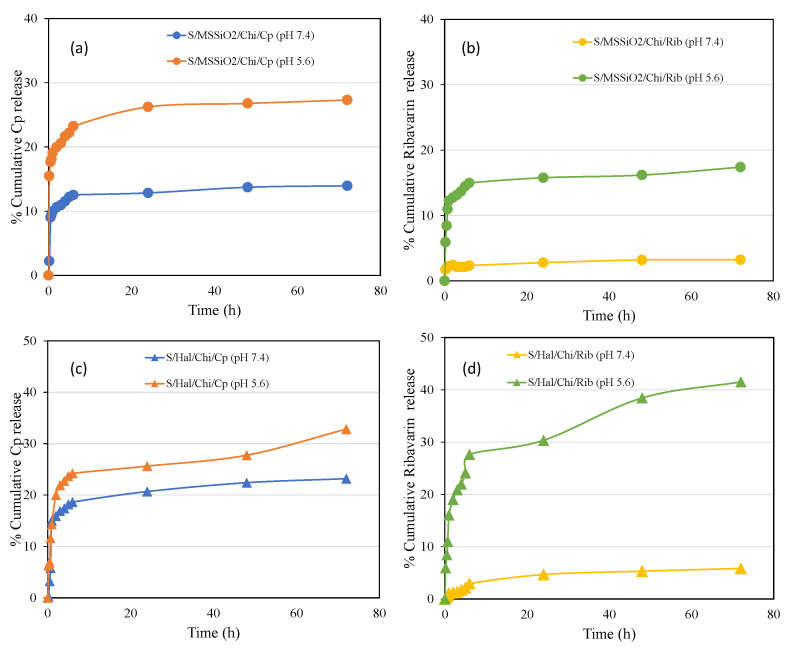
(**a**–**d**) In vitro release profiles of Cp and Rib from S/MSSiO_2_/Chi/Rib/Cp and S/Hal/Chi/Rib/Cp nanoformulations at pH 5.6 and 7.4, respectively.

The pH-responsive release behavior of dual drugs-Cp and Rib was investigated using 10 wt% SPIONs/MSSiO_2_/Rib/Cp/Chitosan and 10 wt% SPIONs/Hal/Rib/Cp/Chitosan over 72 h at two physiologically relevant pH conditions (pH 5.6 and 7.4), as shown in Figure 3. These pH values simulate the acidic tumor microenvironment (pH 5.7) caused by the lactate buildup and other end products [42]. The effect of chitosan, poly(D,L-lactide-co-glycolide), and polyethylene glycol on pH-stimulated drug release, biocompatibility, and mucoadhesion has been extensively investigated [43]. Several studies have reported the development of pH-sensitive drug delivery systems, including dual drug (doxycycline–docetaxel) loaded liposome-targeting folate receptor β [44,45,46]. In the present study, the pH differential between lung cancerous tissues (approximately pH 5–6.8) and normal tissues (pH ~7.4) was exploited as a physiological gradient to facilitate the selective release of Cp and Rib, thereby minimizing off-target effects and reducing systemic toxicity. The MSSiO_2_-based formulation (S/MSSiO_2_/Chi/Rib/Cp) exhibited a drug entrapment efficiency of 91% with a loading capacity of 4.6%, whereas the halloysite-based formulation (S/Hal/Chi/Rib/Cp) demonstrated a slightly lower entrapment efficiency of 88% and a loading capacity of 4.4%. Chitosan served as a pH-responsive wrapping agent around the silica due to its protonable amine groups, which enable enhanced swelling under acidic environments, thereby promoting controlled drug release at tumor-relevant pH levels. Furthermore, the positive charge and mucoadhesive characteristics of chitosan can enhance cellular uptake and facilitate targeted delivery of Cp and Rib [47]. Interestingly, the SARS-CoV-2 virus has been reported to interact with the ACE-2 receptor and fuse into the cell under mild acidic pH conditions between 5 and 6.0 [48], which aligns with the pH range targeted in this study. Additionally, the impregnation of SPIONs enables responsiveness to external magnetic fields, thereby assisting bioimaging as well as improving targeted drug delivery. As shown in Figure 3, Cp (a,c) and Rib (b,d) showed variable release profiles depending on MSSiO_2_-based (a,b) or halloysite-based (c,d) nanoformulations. Overall, the cumulative release percentage of both Cp and Rib in MSSiO_2_^−^ or halloysite-based drug delivery system was higher at pH 5.6 compared to pH 7.4, suggesting a greater release variability in the tumor’s acidic pH than the normal physiological pH. The MSSiO_2_-based formulation released approximately 27% of Cp at pH 5.6, which decreased to 14% at pH 7.4. For Rib, the release was approximately 17% at pH 5.6, which reduced to 3.2% at pH 7.4. In contrast, the halloysite-based drug delivery system exhibited a slightly lesser variability in Cp release between the two pH levels. The cumulative Cp release was 33% at pH 5.6 and 23% at pH 7.4. Notably, Rib release in the halloysite formulation was markedly pH-dependent, with 41% released at pH 5.6 and only 5.8% at pH 7.4. Zarepour et al. [49] previously demonstrated the dual delivery of Rose Bengal and curcumin using a chitosan polymeric shell around the niosomal carrier, wherein the chitosan shell provided pH-responsive behavior via amine protonation. Consistent with their findings, our study confirms that wrapping of chitosan on monodispersed silica effectively mediates pH-sensitive drug release. Moreover, the surface area analysis (Table 1) indicated a high specific surface area in the developed formulations, supporting efficient drug adsorption and diffusion of both Cp and Rib.

**Figure 4:**
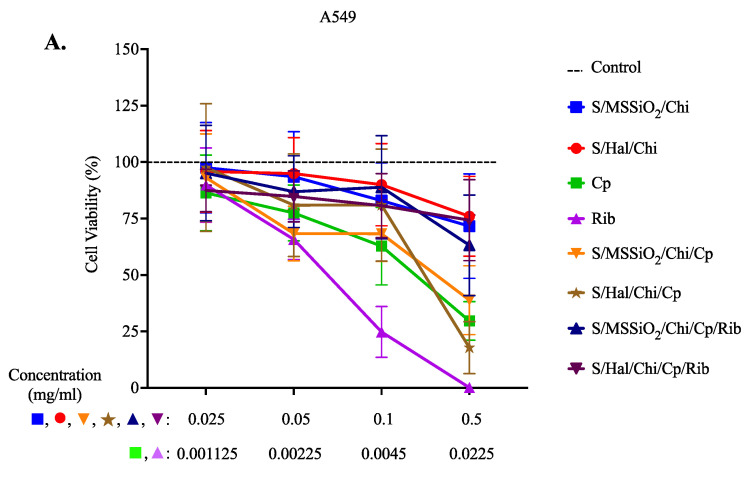

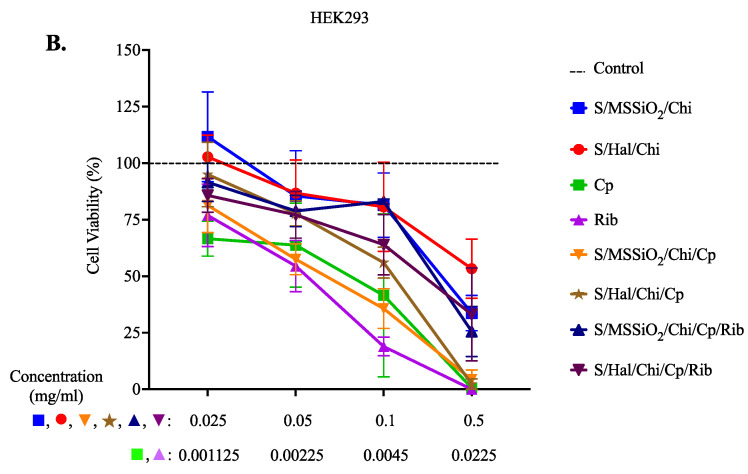
Cell Viability Assay. (**A**) A549 and (**B**) HEK293 cells were treated with S/MSSiO_2_/Chi, S/Hal/Chi, Cp, Rib, S/MSSiO_2_/Chi/Cp, S/Hal/Chi/Cp, S/MSSiO_2_/Chi/Cp/Rib, and S/Hal/Chi/Cp/Rib for 48 h.

We further explored the cytotoxic potential of our nanocomposites using the MTT assay (Figure 4). To dissect the effect of baseline nanoformulations before the addition of cisplatin or ribavirin, we tested the nanocomposites containing superparamagnetic iron oxide (S), monodispersed spherical silica (MMSiO_2_) or halloysite (Hal), and Chitosan (Chi), which are indicated as S/MMSiO_2_/Chi (blue line) and S/Hal/Chi (red line) in Figure 4. In A549 cells, the S/MSSiO_2_/Chi or S/Hal/Chi nanocomposites (blue and red) that did not include Cp or Rib had little effect on viability compared to the no-treatment control. As expected, cisplatin and ribavirin resulted in a significant reduction in cell viability. This is supported by previous studies where the antiviral ribavirin exhibited anticancer activity by inhibiting tumor cell proliferation, reducing angiogenesis proteins, and downregulation of several related proteins such as the ErbB receptor tyrosine kinase receptors [50]. Moreover, cells treated with S/MSSiO_2_/Chi/Cp or S/Hal/Chi/Cp had dose-dependent toxicity similar to the free cisplatin. The optimum concentration range of both formulations S/MSSiO_2_/Chi/Cp or S/Hal/Chi/Cp was found to be between 0.05 and 0.1 mg/mL. Interestingly, the addition of ribavirin (S/MSSiO_2_/Chi/Cp/Rib and S/Hal/Chi/Cp/Rib) resulted in an enhanced cell viability compared to the free cisplatin or ribavirin. On the other hand, HEK293 seems to be more sensitive than the A549 cells, especially after treatment with the S/MSSiO_2_/Chi or S/Hal/Chi nanocomposites and the S/MSSiO_2_/Chi/Cp/Rib or S/Hal/Chi/Cp/Rib treatment groups. Similarly to A549, HEK293 cells treated with S/MSSiO_2_/Chi/Cp/Rib or S/Hal/Chi/Cp/Rib nanocomposites still had a better viability than those treated with free cisplatin and ribavirin. This is more likely due to the low drug release from the chitosan-wrapped nanocomposites at the physiological pH 7.4, which is the pH in cell cultures. As indicated in Figure 3, at pH 7.4 and at 48 h, the cumulative release from the S/MSSiO_2_/Chi/Cp/Rib nanocomposites was 14% for Cp and 3.2% for Rib. Similarly, the cumulative release from the S/Hal/Chi/Cp/Rib nanocomposites was 27% for Cp and 5.9% for Rib.

**Figure 5:**
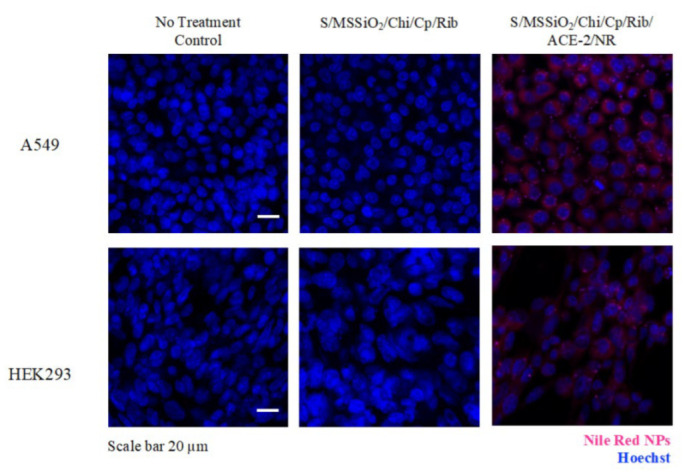
Immunofluorescent imaging of A549 and HEK293 cells treated with S/MSSiO_2_/Chi/Cp/Rib and S/MSSiO_2_/Chi/Cp/Rib/ACE-2/NR at 0.1 mg/mL for 24 h. Scale bar is 20 µm.

Furthermore, we investigated whether the cells were able to successfully internalize our nanocomposite (Figure 5). We functionalized S/MSSiO_2_/Chi/Cp/Rib with ACE-2 and Nile Red (NR). A549 and HEK293 cells were treated with both non-modified and modified nanocomposites (S/MSSiO_2_/Chi/Cp/Rib and S/MSSiO_2_/Chi/Cp/Rib/ACE-2/NR) and then stained with Hoechst. The examination was performed under a confocal fluorescent microscope. The assessment revealed the presence of pink speckles in cytoplasmic compartments of cells treated with the ACE-2/NR-functionalized nanocomposite but not in the cells treated with the non-functionalized version. Currently, the effectiveness of combining platinum-based chemotherapeutic drugs (e.g., cisplatin) and nucleoside antimetabolite drugs (e.g., ribavirin) is gaining interest for antitumor and antiviral effects [51]. In the present study, our results suggest S/MSSiO_2_/Chi/Cp/Rib exhibits successful internalization for potential pulmonary drug delivery treatment of lung cancer and infections.

## 4. Conclusions

The fabrication of a pulmonary drug delivery system remains a significant challenge due to the complexity of the tumor microenvironment and the prevalence of drug resistance. This study investigates the therapeutic potential of a magnetically responsive combinatorial drug delivery system comprising cisplatin, ribavirin, and monodispersed spherical silica nanoparticles for the treatment of lung cancer. Superparamagnetic iron oxide nanoparticles (10 wt% SPIONs) were integrated into a monodispersed spherical silica coated with chitosan (0.6 wt/v%), cisplatin/ribavirin (5% wt/wt), and an ACE-2 receptor (0.5 μL/mL) for the treatment of lung cancer. The coordination nature of SPIONs deposition, surface texture, magnetic saturation, drug and chitosan functionalization, and morphology were analyzed using different physicochemical techniques. It is well known that lung tissue and its cellular physiology are greatly influenced by changes in the pH. The cumulative release studies demonstrated that cisplatin release from S/MSSiO_2_/Rib/Cp/Chitosan nanocomposite reached 33% at pH 5.6, while only 23% was released at pH 7.4. Similarly, ribavirin exhibited a release of 41% at pH 5.6, which decreased sharply to 6% at pH 7.4, confirming an enhanced release profile under acidic conditions. These results, supported by the cytotoxicity assay, suggest that the developed nanoformulations exhibit promising potential for pH-responsive pulmonary drug delivery, with an effective dose range of 0.05–0.1 mg/mL for lung cancer therapy.

## Figures and Tables

**Table 1:** Surface area of nanocarrier and nanoformulations.

Sample	Surface Area (m^2^/g)	Pore Volume (cm^3^/g)	Pore Diameter (nm)
MSSiO_2_	170	0.35	8.3
SPIONs/MSSiO_2_	130	0.28	8.5
S/MSSiO_2_/Chi/Cp/Rib/ACE-2	76	0.28	15

**Table 2:** Statistical analysis of the cell viability assay for A549 and HEK293 cells.

Dose	Treatment Condition	A549 Cells		HEK293 Cells	
		Significant	*p* Value	Significant	*p* Value
**1**	S/MSSiO_2_/Chi (A)	No	0.9999	No	0.9772
	S/Hal/Chi (B)	No	0.9997	No	0.9989
	Cp (C)	No	0.9210	No	0.1408
	Rib (D)	No	0.9722	No	0.5764
	S/MSSiO_2_/Chi/Cp (E)	No	0.9976	No	0.6200
	S/Hal/Chi/Cp (F)	No	>0.9999	No	0.9978
	S/MSSiO_2_/Chi/Cp/Rib (G)	No	0.9997	No	0.8549
	S/Hal/Chi/Cp/Rib (H)	No	0.7081	No	0.4946
**2**	S/MSSiO_2_/Chi (A)	No	0.9986	No	0.9478
	S/Hal/Chi (B)	No	0.9987	No	0.8901
	Cp (C)	No	0.5239	No	0.4849
	Rib (D)	No	0.1735	No	0.1620
	S/MSSiO_2_/Chi/Cp (E)	No	0.3235	No	0.0724
	S/Hal/Chi/Cp (F)	No	0.9112	No	0.1589
	S/MSSiO_2_/Chi/Cp/Rib (G)	No	0.9166	No	0.2530
	S/Hal/Chi/Cp/Rib (H)	No	0.7063	No	0.4116
**3**	S/MSSiO_2_/Chi (A)	No	0.8436	No	0.7216
	S/Hal/Chi (B)	No	0.9835	No	0.8606
	Cp (C)	No	0.4227	No	0.5954
	Rib (D)	No	0.0628	**Yes**	**0.0076**
	S/MSSiO_2_/Chi/Cp (E)	No	0.3279	No	0.0526
	S/Hal/Chi/Cp (F)	No	0.9347	No	0.0676
	S/MSSiO_2_/Chi/Cp/Rib (G)	No	0.9908	**Yes**	**0.0128**
	S/Hal/Chi/Cp/Rib (H)	No	0.6995	No	0.3088
**4**	S/MSSiO_2_/Chi (A)	No	0.7567	**Yes**	**0.0396**
	S/Hal/Chi (B)	No	0.7009	No	0.1973
	Cp (C)	**Yes**	**0.0422**	**Yes**	**0.0001**
	Rib (D)	**Yes**	**<0.0001**	**Yes**	**<0.0001**
	S/MSSiO_2_/Chi/Cp (E)	No	0.1614	**Yes**	**0.0059**
	S/Hal/Chi/Cp (F)	No	0.0555	**Yes**	**0.0017**
	S/MSSiO_2_/Chi/Cp/Rib (G)	No	0.5827	No	0.0624
	S/Hal/Chi/Cp/Rib (H)	No	0.6693	No	0.2299

## Data Availability

The original contributions presented in this study are included in the article. Further inquiries can be directed to the corresponding authors.
